# Sphenoid Bone Fibrous Dysplasia Detected Incidentally on Bone Scintigraphy by the Contribution of SPECT/CT Hybrid Imaging

**DOI:** 10.4274/mirt.93685

**Published:** 2018-02-01

**Authors:** Hüseyin Şan, Kürşat Okuyucu, Ali Ozan Öner, Özdeş Emer, Alper Özgür Karaçalıoğlu

**Affiliations:** 1 Karabük Training and Research Hospital, Clinic of Nuclear Medicine, Karabük, Turkey; 2 Gülhane Training and Research Hospital, Clinic of Nuclear Medicine, Ankara, Turkey; 3 Afyon Kocatepe University Faculty of Medicine, Department of Nuclear Medicine, Afyon, Turkey

**Keywords:** Craniofacial fibrous dysplasia, technetium-99m-methylene diphosphonate bone scintigraphy, single-photon emission computed tomography/computed tomography

## Abstract

Fibrous dysplasia (FD) is a benign fibroosseous bone disorder. It has poliostotic and monostotic patterns. Monostotic FD is frequently asymptomatic and is usually discovered incidentally by radiologic imaging performed for other reasons. Bone scintigraphy is valuable for identifying disease extent. Craniofacial FD (CFD) is a form of the disease where lesions are limited to contiguous bones of the craniofacial skeleton. We presented a case with monostotic CFD who was detected incidentally on bone scintigraphy single-photon emission computed tomography/computerized tomography while being investigated for inflammatory arthropaties.

## Abstract (tr)

Fibröz displazi (FD) kemiklerin iyi huylu bir fibroosseöz bozukluğudur. Poliyostatik ve monostatik formları vardır. Monostatik FD sıklıkla asemptomatiktir ve genellikle radyolojik görüntülerde tesadüfen fark edilir. Kemik sintigrafisi hastalığın yayılımını belirlemede değerlidir. Kraniyofasiyal FD (CFD) hastalığın kraniyofasiyal iskeletin kemiklerine sınırlı olduğu bir formudur. Enflamatuvar artropati yönünden araştırılırken tesadüfen kemik sintigrafisinde tek-foton emisyon bilgisayarlı tomografi/bilgisayarlı tomografi ile tespit edilen bir monostatik CFD olgusunu takdim ediyoruz.

## Figures and Tables

**Figure 1:**
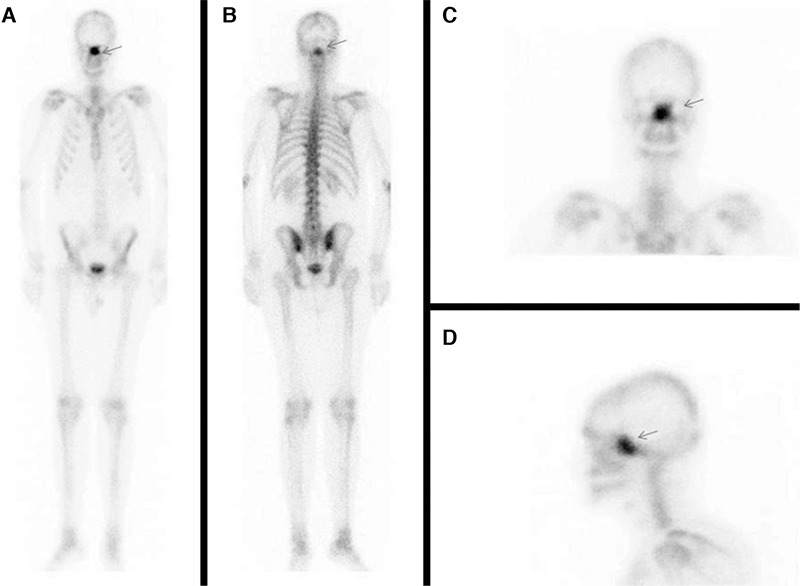
Anterior whole-body (A), posterior whole-body (B), anterior head spot (C), left lateral head spot (D) late static planar images of technetium (Tc)-99m-methylene diphosphonate (MDP) bone scintigraphy showing uptake in the middle facial region (arrows). Fibrous dysplasia (FD) is a benign disorder of the bone in which there is developmental arrest of all components of normal bone (1). The lesions progressively replace the medullary cavity (2). Long bones, ribs, craniofacial bones and pelvis are the most common sites of skeletal involvement (3). It has been reported that the monostotic form constitute 70% of cases while 30% are polyostotic (4). Craniofacial FD (CFD) is a form of the disease where lesions are limited to contiguous bones of the craniofacial skeleton (5,6). Craniofacial involvement is present in 10-27% of monostotic cases and 50% of polyostotic ones (7,8). CFD without involvement of bones out of the cranium can not be easily described as monostotic because of the potential adjoining involvement of cranial bones (6,9). A 20-year-old male with back pain was investigated for seronegative spondylarthropathy. In order to demonstrate any inflammatory joint involvement, a three-phase Tc-99m-MDP bone scintigraphy was requested. Although perfusion and blood pool phases were normal, there was intense MDP uptake in the middle facial region on late static phase whole-body images.

**Figure 2:**
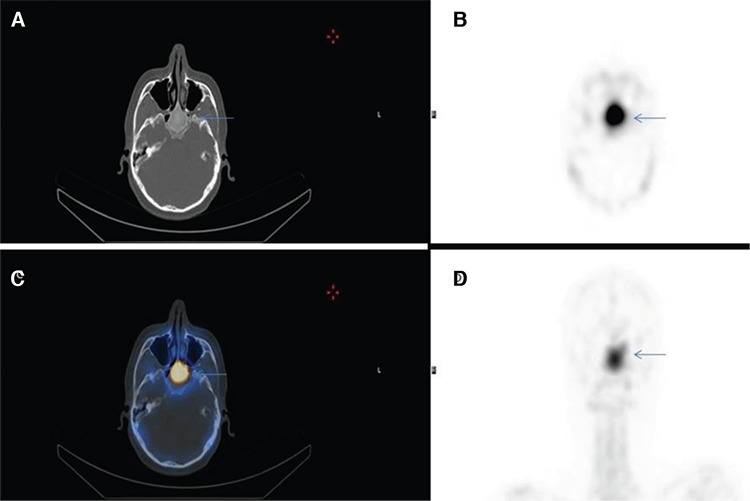
Computerized tomography (CT) (A), single-photon emission CT (SPECT) (B), fusion (C), maximum intensity projection (D) images on SPECT/CT hybrid imaging of Tc-99m-MDP bone scintigraphy that localize and spot the uptake on planar images in the sphenoid bone (arrows). For the exact localization of this abnormal incidental finding, SPECT/CT hybrid imaging was performed. This accumulation was situated at and confined to a well-marginated, ground-glass opacity in the corpus of the sphenoid bone on CT component (Figure 2). The lesion was resembling a benign bone disorder (suggestive of monostotic FD). Magnetic resonance imaging (MRI) was requested to differentiate it from other benign bone pathologies that confirmed the diagnosis of FD. CT is the best technique for depicting lesion extent, cortical boundary and homogeneity of the poorly mineralized lesion (10). Well margination, hazy ground-glass opacity and contrast enhancement are characteristic features of FD on CT (10,11). MRI is quite sensitive for detecting FD and provides complementary information to CT (12). Although bone scintigraphy has low specificity for FD, it has a valuable role on identifying disease extent at initial presentation due to its high sensitivity (10,11). Despite its high capability of lesion detection, determining exact lesion location by this method is problematic especially for contiguous bones of the craniofacial region. CT and MRI are good options to overcome this obstacle easily, by providing accurate anatomical detail. On the other hand, without a whole-body imaging which is not practical by CT or MRI, whether CFD is polyostotic or not can not be clarified. For this reason, existence of any lesion in other parts of the body other than the craniofacial region should be clearly depicted. A whole-body bone scintigraphy at a single session and an additional SPECT/CT, which provides both anatomical and functional data, will be sufficient to elucidate this issue.
